# Gynecological Cancers in Lynch Syndrome: A Comparison of the Histological Features with Sporadic Cases of the General Population

**DOI:** 10.3390/jcm11133689

**Published:** 2022-06-27

**Authors:** Valentina Elisabetta Bounous, Elisabetta Robba, Stefania Perotto, Barbara Pasini, Nicoletta Tomasi Cont, Maria Teresa Ricci, Antonino Ditto, Marco Vitellaro, Francesco Raspagliesi, Nicoletta Biglia

**Affiliations:** 1 Academic Division of Obstetrics and Gynecology—A.O. Ordine Mauriziano, University of Turin, 10128 Turin, Italy; elisabetta.robba@gmail.com (E.R.); nicoletta.biglia@unito.it (N.B.); 2 Ospedale Michele e Pietro Ferrero, 12060 Verduno, Italy; stefania.perotto@gmail.com; 3 Department of Genetics, Biology and Biochemistry, University of Turin, 10128 Turin, Italy; barbara.pasini@unito.it; 4 ASL TO 3, 10093 Collegno, Italy; nicolettatomasi@tiscali.it; 5 Unit of Hereditary Digestive Tract Tumors, Fondazione IRCCs—National Cancer Institute, 20133 Milan, Italy; mariateresa.ricci@istitutotumori.mi.it (M.T.R.); marco.vitellaro@istitutotumori.mi.it (M.V.); 6 Division of Gynecologic Oncology, Fondazione IRCCs—National Cancer Institute, 20133 Milan, Italy; antonino.ditto@istitutotumori.mi.it (A.D.); francesco.raspagliesi@istitutotumori.mi.it (F.R.)

**Keywords:** Lynch syndrome, endometrial cancer, ovarian cancer, mismatch repair genes

## Abstract

Introduction: About 5% of endometrial cancers (ECs) are attributed to an inherited predisposition, for which Lynch syndrome (LS) accounts for the majority of cases. Women with LS have a 40–60% predicted lifetime risk of developing EC, in addition to a 40–80% lifetime risk of developing colorectal cancer and other cancers. In this population, the lifetime risk of developing ovarian cancer (OC) is 10–12%. Object: to compare the histopathological features of LS-associated EC and OC with sporadic cancers in order to evaluate whether there are differences in terms of age at diagnosis, site of occurrence in the uterus, histological type, stage at diagnosis, and tumor grading. Materials and methods: we compared data obtained from 96 patients with LS-associated gynecological cancers (82 with EC and 14 with OC) to a control group (CG) of 209 patients who developed sporadic EC, and a CG of 187 patients with sporadic OC. Results: The mean age at diagnosis of LS-associated EC and OC was much lower than in the control groups. In both groups with EC, the endometrioid histotype was the most frequently occurring histotype. However, among LS women there was a significantly higher incidence of clear cell tumors (11% versus 2.4% in the CG, *p* = 0.0001). Similar to the sporadic cancer cases, most of the LS-associated ECs presented at an early stage (89% of cases at FIGO I-II stage). In the LS group, the tumor frequently involved only the inner half of the endometrium (77% of cases, *p* < 0.01). In the LS group, 7.3% of ECs were localized to the lower uterine segment (LUS), whereas no cancer developed in the LUS in the CG. No serous OCs were diagnosed in the LS group (versus 45.5% in the CG, *p* = 0.0009). Most of the LS-associated OCs presented at an early stage (85% of cases at FIGO I-II stages, *p* < 0.01). Conclusion: LS-associated EC and OC seem to have peculiar features, occurring at a younger age and at an earlier stage. In LS, EC less frequently involves the outer half of the endometrium, with a more frequent occurrence in the LUS. The presence of clear cell EC was more frequently observed, whereas in OC, the predominant histotype was endometrioid.

## 1. Introduction

Lynch syndrome (LS), also known as Hereditary Non-Polyposis Colorectal Cancer syndrome (HNPCC), is an autosomal dominant inherited cancer susceptibility syndrome with a medium to high degree of penetrance (30–70%). It is caused by a germline mutation in one of the genes in the DNA mismatch repair gene (MMR) family, which includes MSH2, MLH1, MSH6, and less commonly PMS1 and PMS2. The prevalence of this syndrome is 0.9 up to 2.7% [1].

LS is associated with a very high risk of developing colorectal cancer, which is typically diagnosed at an early age and with a proximal colonic predilection; 70–85% of colorectal cancers in LS are next to the splenic flexure. According to the literature, the lifetime risk of colorectal cancer ranges from 25–83% in females [2]. LS is associated with an elevated risk of multiple extracolonic cancers, including cancer of the endometrium, ovary, stomach, small bowel, hepatobiliary tract, transitional cell carcinoma of the ureter and renal pelvis, brain, and skin tumors of the Muir-Torre Syndrome [3].

LS accounts for approximately 3% of all colon cancers and may account for a similar number of ECs. The LS phenotypes include a propensity for cancers of the proximal colon, poor tumor differentiation with mucinous or signet-ring cell histologic features or a medullary growth pattern, abundant infiltrating lymphocytes in the tumor, and synchronous and metachronous colorectal cancers [4].

For patients with LS, the lifetime risk of developing endometrial cancer (EC) varies from 30 to 70%, and the lifetime risk of developing ovarian cancer (OC) is 12–15% [5].

Extracolonic cancers are more often observed in MSH2 mutations compared to MLH1 mutation families [6,7]. The risk of developing EC and OC in LS varies depending on which gene has mutated, as described by the Prospective Lynch Syndrome Data base (PLSD) [8], which reported an incidence of EC and OC at 75 years of age of 37% and 11% for MLH1 carriers, 49% and 17% for MHS2 carriers, and 41% and 11% for MSH6 carriers, respectively [9]. Ovarian cancer, particularly of the endometrioid type, is related to MLH1 mutations [10]. The synchronous diagnosis of an endometrioid EC and an endometrioid OC is a relatively common situation observed in LS [11].

Furthermore, women with a mutation in the MSH6 gene probably have a milder clinical phenotype with a later onset of both colorectal cancer and EC [12,13]. The lifetime risk for women with a mutation in the PMS2 gene is unknown, but studies have suggested that these patients have a milder phenotype compared to women with mutations in the MLH1 and MSH2 genes [14,15,16]. 

In more than half of the mutated patients with metachronous colorectal and gynecological cancers, EC or OC are the tumors that are diagnosed first, making these the “sentinel cancers” of the syndrome [17]. Before DNA mismatch repair gene mutations were used to determine germline genetic defects in families with LS, clinical criteria (called Amsterdam criteria) based on an early age at cancer onset and the presence of more cancers among family members, defined individuals with LS [18,19]. Similar criteria were included in the Bethesda Guidelines that were developed in 1997 and revised in 2004 [20]. If the Amsterdam Criteria or Bethesda Guidelines are met, molecular pathology testing of the cancer for alterations typical of LS is indicated. This includes testing for microsatellite instability (MSI) and MMR protein immunohistochemistry (IHC). Although the Amsterdam criteria is commonly used, it has poor sensitivity for the detection of LS, which is often underdiagnosed [21].

The diagnosis of LS allows clinicians to tailor treatment and clinical management, and to optimize counselling and cancer surveillance for patients and their families. With regards to surveillance for colon cancer, the early detection of lesions by colonoscopy is associated with improved survival [8]. For gynecological cancers, there is no clear data that points to the benefit of surveillance on survival [22]. However, multicenter studies assessing the benefits of surveillance in asymptomatic women with LS, considering their age, menopausal state, and surveillance interval, needs to be performed. Most recommend the use of transvaginal ultrasound and endometrial biopsy for premenopausal women, and transvaginal ultrasound alone for asymptomatic postmenopausal women [23].

In contrast, risk-reducing hysterectomy and bilateral salpingo-oophorectomy have been shown to prevent gynecological cancer in women with LS, and should be recommended following the completion of childbearing for MLH1, MSH2, and MSH6 carriers over 35–40 years of age [8].

Regarding outcomes, a favorable prognosis was suggested, which was probably related to the active local immune response. The release of peptides by MMR-deficient tumors allows the patients’ immune systems to better recognize them [24].

Colorectal cancers with deficient mismatch repair are associated with an earlier stage at diagnosis, a lower propensity for metastasis, and consequently a significantly better prognosis than patients with stage-matched cancers with proficient mismatch repair [4]. With regards to EC, the new prognostic molecular classification introduced by The Cancer Genome Atlas Research Network (TCGA) suggests a better prognosis for MSI hypermutated EC compared to TP53 tumors [25]. Due to its prognostic role, MMR status has been recently added to EC management guidelines and is fundamental for the post-surgical treatment of early-stage EC. Due to its prognostic role, MMR status should be performed for all ECs, irrespective of the histologic subtype and of the patient’s age [26]. 

As in ECs, deficient MMR endometrioid OCs are associated with better prognoses compared to those with mutated TP53 genes, according to some reports [11].

Concerning treatment, new promising drugs specifically acting on MMR-deficient tumors have been evaluated. In particular, in 2017 the anti-PD-1 immune checkpoint antibody pembrolizumab was introduced for advanced MMR-deficient cancers, being the the first anti-neoplastic agent to be given a site agnostic license since it can be prescribed irrespective of the tumor site and depends only on the presence of MMR deficiency [27]. Furthermore, for this reason, the identification of MMR-deficient tumors is so important for patient care [24].

Although there are several epidemiologic studies of ECs and OCs in women with LS, detailed pathological analyses are lacking. It is not known with certainty whether ECs and OCs have unique pathological features in women with LS compared with sporadic tumors. Previous studies have suggested that in LS, EC is associated with a diagnosis at an earlier age [28] and stage, and ECs have different histology compared with the general population [29,30]. Moreover, ECs associated with LS are thought to be preferentially located in the lower segment of the uterus, but not for MLH1-mutated ECs. With regards to OC, a higher incidence of non-serous tumors diagnosed in LS women was observed [31].

The knowledge generated from these data is critical for understanding the natural history of EC and OC in this unique population of patients.

Therefore, the primary purpose of this study was to examine the pathological features of EC and OC in women with LS compared to sporadic cancers.

## 2. Materials and Methods

In our study we included 96 patients with ascertained LS, of which 82 were diagnosed with EC and 14 with OC. Data were collected in the databases of the departments of Medical Genetics of Molinette Hospital of Turin, Medical Genetics of San Luigi Gonzaga Hospital of Orbassano (Turin), Medical Genetics of IRCCs Candiolo Hospital, and IRCCs INT of Milan. Data related to diagnosis and treatment were found in the archives of the above institutions. The LS patients included in our study had genetic counselling because their personal or family history was suggestive of LS. The diagnosis was confirmed by observations of microsatellite instability and by IHC analysis. Pathologists classified the tumors according to the WHO (World Health Organization). Because of the study period, the grade and stage were defined according to the 1988 FIGO classification for OC and the 2009 FIGO classification for EC [32,33].

In the control group, we included 209 patients with EC and 187 patients with OC who did not have a family history suggestive of cancer, and who underwent surgery between 2006 and 2016 in the department of Gynecology and Obstetrics of the Umberto I Hospital of Turin. The women selected in the control group met the following inclusion criteria: a clinical and histopathological diagnosis of EC, or a clinical and histopathological diagnosis of OC at any age. Exclusion criteria included a definitive histological diagnosis of benign endometrial or ovarian pathology, a diagnosis of simple, typical complex, or atypical complex hyperplasia, and a family history of EC, OC, and/or colorectal cancer (to rule out the inclusion of possible mutation carriers, although they were untested for MMR gene mutations).

### Statistical Analysis

Statistical analysis was performed using Fisher’s exact test and Chi-square tests (X2). Fisher’s exact test and X2 were used to compare and assess the significance of differences between the two groups (LS and the CG) for categorical variables (histological features). Continuous variables, such as the mean age at diagnosis of EC and OC in different groups, were analyzed using *t* tests. *p* values were two sided and *p* < 0.05 was considered significant for X2 and Fisher’s exact tests, whereas *p* < 0.01 was significant for *t* tests. Data were analyzed using the Statistical Package for Social Science (SPSS) version 24.0 (IBM, Armonk, NY, USA).

## 3. Results

### 3.1. Endometrial Cancer 

The mean age for diagnosis of EC in women with LS was 48.5 years (with a range of 30–78 years), which was significantly lower than the control group (67 years, with a range of 37–90 years; *p* < 0.01) (Table 1). In the LS group, 59% of patients received a diagnosis before 50 years of age (4.3% in general population). 

In LS, a younger age was reported for mucinous histology (mean age at onset: 35 years) and an older age was reported for women with endometrioid and clear cell EC (53 years). 

For EC in LS patients, a mutation in the MSH2 gene occurred more frequently (48.8%), followed by a mutation in the MLH1 gene (42.6%) and in the MSH6 gene (8.6%) (Table 2). 

With regards to EC histotype (Table 3), in the LS group, the majority of ECs were of endometrioid histotype (78% versus 88% for the control group; *p* = 0.0635). A significantly higher proportion of clear cell EC was noted among the LS-associated tumors (11% versus 2.4% for the control group, *p* = 0.0001). In both groups, the most frequent site of EC was the corpus uteri, however a lower incidence was observed in LS patients (73.2% versus 91.9% in the control group; *p* = 0.0001). Moreover, the second most frequent site of occurrence of EC in LS was the uterine fundus (15.8% versus 4.8% in the control group; *p* = 0.0016). Interestingly, occurrence in the lower uterine segment (LUS) was noticed more frequently in LS patients (7.3% in LS versus 0% in the control group; *p* = 0.0001) (Table 3). 

Regarding the grading, there were no statistically significant differences between the two groups. LS-associated EC shows a trend towards a higher prevalence of moderately differentiated carcinomas (G2); this is similar to the general population (42.2% versus 41.5% in the control group; *p* = 0.9744). Furthermore, LS-associated EC has a lower incidence of well-differentiated cancer G1 (30% versus 21.1% in the control group; *p* = 0.1286). 

Compared to EC in the control group, invasion by LS tumors is more frequently limited to the inner half of the myometrium (76.8% versus 36.7% in sporadic EC; *p* < 0.0001). 

In both the LS group and the control group, EC is diagnosed more often at FIGO stage I (72% versus 70% in the control group; *p* = 0.6101) (Table 4).

### 3.2. Ovarian Cancer

The mean age at diagnosis of OC in LS patients was 45 years (with a range of 32–78 years), which is significantly lower than in the control group (58 years, with a range of 31–86 years; *p* < 0.01). In the LS group, 64.3% of patients developed OC before 45 years of age (Table 5).

The most frequent mutation in OC patients was in the MSH2 gene (57%), followed by the MLH1 gene (43%). In our study, none of the women with a diagnosis of OC reported an MSH6 or PMS2 gene mutation (Table 6).

In our analysis, the endometrioid histotype was diagnosed in 36% of LS patients and in only 21% of the control group with OC, however this difference was not significant (*p* = 0.1036). On the contrary, in the control group, serous OC was most frequently diagnosed (45.5% of cases). Interestingly, this was not found among LS patients (*p* = 0.0009). The incidence of clear cell carcinoma was significantly higher in women with LS (29% versus 5.8% in the control group, *p* = 0.0018) (Table 7).

The data concerning grading suggested that, in the general population, OCs were mostly poorly differentiated—G3-(81.5%); on the contrary, only 50% of OCs among LS patients were G3 (*p* = 0.0056).

In our analysis, LS women received a diagnosis of OC at an earlier stage than in the general population. Among patients with LS-related OC, 71.4% were diagnosed in stage I and 14.4% in stage II; this was significantly different from the control group (25%, *p* = 0.0001 and 4%, *p* = 0.0819, respectively). On the contrary, OC tumors in the control group were more frequently diagnosed at an advanced FIGO stage (57% stage III versus 7.1% in the LS group; *p* = 0.0003).

## 4. Discussion

Data regarding the features of LS-related EC and/or OC are limited. The aim of this study was to evaluate the clinical and pathological features of EC and OC diagnosed in patients with LS compared to the gynecological cancers observed in the control group, which are representative of the general population. A strength of the study is in its multicenter design. The study is limited by the lack of available data regarding both personal and family histories of cancers other than EC/OC.

### 4.1. Endometrial Cancer

EC is generally diagnosed at an early stage with a low recurrence rate. However, there remains an urgent clinical need to identify high-risk patients in order to ensure tailored treatment [26].

LS-associated EC represents the most common extraintestinal sentinel cancer of LS, which indicates a risk for the subsequent development of other tumors. Therefore, there is a need for screening and preventive strategies in order to decrease cancer-related morbidity and mortality [23].

In our study, LS women frequently carried MSH2 gene mutations. We reported this mutation in 48.8% of LS women, which was followed by MLH1 gene mutations (42.6%). These data are in line with literature concerning the frequency of the different mutations of the mismatch repair genes [5,34,35,36]. Significantly, there was a higher incidence, in our cases, of mutations in the MSH6 gene (8.5%). Many studies have reported a prevalence of 6% in people with LS. Mutations of this gene seem to be associated with milder disease and a better prognosis. However, compared to MLH1 and MSH2 gene mutations, this confers a higher risk of developing EC of up to 71% [7,12,13,16]. Moreover, it is reported in the literature that mutations in MSH6 genes are associated with an onset of EC at an older age compared to mutations in MSH2 or MLH1 genes [37]. Women with an MSH6 mutation have a 26-fold increased risk of EC and 6-fold increased risk of cancer related to LS. In line with these results, the risk of EC in older women with MSH6 mutations is estimated to be 26–44-fold [12].

The cumulative risk of EC in women aged 75 years with MLH1, MSH2, MSH6, or PMS2 mutations is estimated to be 37%, 48.9%, 41.1%, and 12.8%, respectively [8].

In patients with LS, the mean age at diagnosis of EC is, in accordance with the literature, lower than in the general population [28]. ECs, in our study, developed about 18 years earlier than sporadic tumors, with a mean age at diagnosis of 48.5 years compared to 67 years in the control group. In the study conducted by Garg, ref. [38] patients with genetic mutations developed EC at an even lower age (37 years) compared to the patients in our study. On the contrary, Rabban et al. [39] reported an older age at onset of EC in LS patients (64 years). Moreover, different mutations resulted in differences in the age at onset; a study reported that MSH6 carriers developed EC at later ages than MSH2 or MLH1 carriers. The range of ages at EC diagnosis were found to be 50.6–59.5 years in MSH6 and 39–49.5 years in MSH2/MLH1 [37].

In a study by Johnallty et al., the strongest predictors of EC were the close relatedness and younger age at EC diagnosis in one or more relatives [40]. The same results were reported in a French multicenter study on 49 patients with LS-associated EC, with a mean age at diagnosis of 49.7 years [41].

With regards to tumor histotype, our data are in line with most of the literature, which states that endometrioid EC is the type of cancer diagnosed in 78% of LS women [13,42,43]. Broaddus et al. observed an endometrioid histological type in 86% of LS-associated EC (versus 78% in the general population), and in 14% of non-endometrioid subtypes (versus 22% in the general population) (*p* = 0.006) [29]. In the French study, the endometrioid histotype accounted for 89.2% of LS-associated EC [41]. On the contrary, Carcangiu et al. reported different results; they reported a higher incidence (43.4%) of non-endometrioid EC, of which 21.7% was clear cell EC and 8.7% was serous-papillary EC [44]. Women with an MSH2 mutation exhibit more non-endometrioid tumors, resulting in a more variable histological spectrum of LS-associated EC [29].

In our study, ECs in both the control group and the LS group were diagnosed more frequently at stage I. Our analysis confirms the results reported in previous studies [13,42]. Regarding tumor grade, we observed a non-significant trend towards a higher prevalence of moderately differentiated G2 tumors in women with LS. In the literature, conflicting results are reported. In one study, the majority of LS-associated ECs were poorly differentiated; among the sporadic carcinomas, 12.1% were well-differentiated and 74.1% were moderately differentiated [44]. Other studies have reported a greater frequency of G1 or G2 EC in women with LS [43]. Rossi found that grade 3 EC occurred only in 19.3% of cases [39]. Soliman et al. found a greater incidence of grade 2 EC (46%), followed by G1 (42%), and G3 EC (12%) [45].

A relevant finding was the significantly higher incidence in carcinomas of the LUS among women with LS (7.3% versus 0% in the control group). LUS is a site that seems to be strongly associated with LS. Other studies found a similar proportion of tumors located in this site [30,46], and in the French study, the LUS was involved in 25% of the cases of EC that occurred in patients with proven LS [41]. Westin et al. found that 14.2% of LS-associated EC involved the LUS, whereas 1.8% of the cases diagnosed in the general population involved the LUS [30]. Another study found that the incidence of LS-associated EC in the LUS was 11.1% [46].

According to the data collected in our study, ECs in women with LS were significantly more frequently confined to the inner half of the myometrium (in 76.8% of the cases versus 37.3% of the control group). Other studies have reported similar results. This finding may explain, at least in part, the increased survival reported for LS patients [42,45].

LS-associated EC may have a better outcome [47]. Since the dualistic model is no longer able to classify EC in a correct way, the new molecular classification introduced by The Cancer Genome Atlas Research Network (TCGA) added prognostic and predictive information, giving us the ability to identify high-risk patients [25]. For this reason, molecular classification including MMR status has been added to the most recent guidelines for EC management, which is relevant for post-surgical treatment in the early stages of EC. The guidelines state that MMR status should be performed for all ECs, irrespective of the histological subtype and of the patient’s age. Since it has a prognostic role, it identifies patients who have a high risk of developing LS and it can be used as a predictor of the potential utility of immune checkpoint inhibitor therapy [26].

With regards to the increased neoantigens induced by higher mutations, tumors with MSI or a deficiency in MMR have also been suggested to be related to a higher presence of tumor-infiltrating lymphocytes (TILs), which may improve a patient’s prognosis [48].

Current guidelines recommend the universal screening of CRC for LS. Because similar rates of LS are observed in EC and CRC, and because of the possibility of reducing mortality through colorectal screening and cascade testing in relatives, the Consensus Group strongly recommends surveillance and genetic testing for women with EC [22].

### 4.2. Ovarian Cancer

LS is related to various histological types of OC, whereas high-grade serous carcinoma is the main histological type of hereditary OC and is related to mutations in the BRCA gene. These data reveal that OC may have different features in BRCA-mutated patients and in patients with LS [49]. The percentage of OC with MMR deficiency is between 2 and 10% of cases, but reaches 20% in endometrioid tumors [11].

Depending on the particular MMR gene, LS increases the cumulative lifetime risk of OC from 6% to 12% [8].

In our study, OC was diagnosed 13 years earlier in patients with LS than in the control group, with a significantly younger age at diagnosis (45 years in the LS group compared to 58 years in the control group). Other studies reported similar findings: the mean age at onset ranges from 42 to 49 years in LS patients in different studies, whereas in the general population, the mean age at onset is 60–65 years [5,37,38,39,40]. A systematic review of 40 studies reported that the average age at OC diagnosis was 45.3 years. The most frequent mutations were in the MLH1 (38%) and MSH2 (47%) genes [50].

In our study, we found a significant association between the presence of the MSH2 gene mutation and the development of OC (57%) [51,52].

The distribution of the histological types of OC among LS patients in our study differs considerably from the general population. Endometrioid OC represented 36% of tumors and clear cell cancer represented 29% of tumors, compared to approximately 21.4% and 5.8%, respectively, in the general population. Several studies reported a wide variety of OC associated with LS, such as clear cell carcinomas, mucinous, endometrioid tumors, and mixed-type carcinomas [35]. In a study on 53 cases of OC in LS patients, the predominant histological subtype was endometrioid (53% of cases) and most cases presented with early stages at onset (85% at stage I and II) [51]. In a retrospective study conducted by Watson et al., 94% of epithelial tumors (including serous, endometrioid, clear cell carcinoma, and mixed-type) and 6% of non-epithelial OCs were observed in LS patients [52]. Crijnen et al. reported non-serous OC in 37% of cases [53], and Watson and Lynch reported this type of tumor in 65% of LS patients [7]. A review reported a high incidence of mixed-type OC (mucinous, endometrioid, or clear cell carcinoma) equal to 31%, followed by endometrioid (23%), serous (21%), and clear cell OC (11%); as described in smaller studies, almost 80% of OC cases were of a non-serous histotype [50].

Woolderink et al. reported endometrioid adenocarcinoma (40%) and serous carcinoma (36%) more frequently, with a very high rate (87%) of tumors diagnosed at an early stage FIGO I/II [54].

MSI occurs in a limited percentage of OC (2–20%) and affects predominantly endometrioid (19.2%), mucinous (16.9%), clear cell (11.2%), and serous (7.9%) histotypes. Both endometrioid and clear cell OC with MSI presented increased levels of TILs, and thus may be susceptible to immune checkpoint inhibitor monotherapy [55].

In our study, the majority of OC in LS patients were well-differentiated or moderately differentiated and were FIGO stage I or II at diagnosis. On the contrary, sporadic OCs are diagnosed at advanced stages in 71% of cases. Similar data were reported in a large-scale analysis conducted by Watson et al., who found that 61% of cases associated with LS were at stage I, 23% were at stage II, 14% were at stage III, and only 2% were at stage IV [52]. In a systematic review, 65% of women were diagnosed at FIGO stage I/II and 18% at FIGO stage III/IV, whereas 17% of cases were unknown [50]. More than two-thirds of sporadic OCs present at an advanced tumor stage, whereas tumors associated with LS typically present at an earlier stage, with 35% of cases presenting as stage I neoplasms [56]. 

## 5. Conclusions

The lifetime risk of EC and OC is increased in patients with LS. EC and OC associated with LS occur at an earlier age than sporadic cancers, are predominantly present at an early stage, with a well or moderate grade of differentiation, and are often of endometrioid or clear cell histology.

The evaluation of a patient’s MMR status is becoming essential for all ECs because of its prognostic and therapeutic role.

There is a general consensus that healthy women with LS should be offered a risk-reducing prophylactic hysterectomy and bilateral salpingo-oophorectomy at around 40 years of age. Despite EC screening, the utility of OC surveillance in a healthy population is not yet evidence-based, nor is it evidence-based in patients with LS, in BRCA-mutated patients, or in the general population. Surveillance can be tailored to individual women. Recognition of these features and appropriate genetic testing enables the identification of gynecological cancers associated with LS, thereby allowing for tailored surveillance, treatment, or surgical prevention.

## Figures and Tables

**Table 1:** Age at diagnosis of endometrial cancer for LS patients and the control group.

Endometrial Cancer	Lynch Syndrome	Control Group	*p*
Mean age (years, ys)	48.9	67	*p* < 0.001
Range (years, ys)	28–78	37–90	
Median age (years, ys)	48	67	*p* < 0.001

**Table 2:** Frequency of MMR gene mutations in LS-related EC.

Mutation	N	%	Mean Age (Years, ys)
hMLH1	35	42.6	53.1 (41–78)
hMSH2	40	48.8	43.5 (30–62)
hMSH6	7	8.5	55.4 (48–61)

**Table 3:** Histological type and onset site of EC in the LS group and in the control group.

		LS (N, %)	Control Group	* p *
Histological type	Endometrioid	64 (78%)	184 (88%)	* p * = 0.0635
	Clear cells	9 (11%)	5 (2.4%)	* p * = 0.0001
	Serous-papillary	8 (9.8%)	13 (6.2%)	* p * = 0.2943
	Mucinous	1 (1.2%)	7 (3.4%)	* p * = 0.3175
Onset site	Corpus	60 (73.2%)	192 (91.9%)	* p * = 0.0001
	Fundus	13 (15.8%)	10 (4.8%)	* p * = 0.0016
	LUS	6 (7.3%)	0	* p * = 0.0001
	Cervix	3 (3.7%)	7 (3.3%)	* p * = 0.8963

**Table 4:** FIGO (International Federation of Gynecology and Obstetrics) stage at diagnosis of EC in the LS group and in the control group.

FIGO	Lynch Syndrome (N, %)	Control Group	*p*
I	59 (72%)	146 (70%)	*p* = 0.6101
II	14 (17%)	22 (10.5%)	*p* = 0.0974
III	8 (9.8%)	35 (16.7%)	*p* = 0.1306
IV	1 (1.2%)	6 (2.8%)	*p* = 0.4082

**Table 5:** Age at diagnosis of Ovaraian Cancer for Lynch Syndrome patients and the control group.

OC	LS	Control Group	*p*
Mean age (years, ys)	45.6	58	*p* < 0.001
Range (years, ys)	32–78	31–86	
Median age (years, ys)	42	60	*p* < 0.001

**Table 6:** Frequency of MMR gene mutation in LS-related OC.

Mutation	N	%	Mean Age [Range]
hMLH1	6	42.9%	50.1 (38–78)
hMSH2	8	57.1%	41.1 (32–52)

**Table 7:** Histological type of OC in the LS group and in the control group.

Histological Type	LS	Control Group	*p*
Serous	0	85 (45.5%)	*p* = 0.0009
Endometrioid	5 (36%)	40 (21.4%)	*p* = 0.1036
Clear cell	4 (29%)	11 (5.8%)	*p* = 0.0018
Serous-papillary	2 (14%)	3 (1.6%)	*p* = 0.0033
Mucinous	2 (14%)	2(6.7%)	*p* = 0.3853
Undifferentiated	1 (7%)	18 (9.6%)	*p* = 0.7594
Borderline tumor	0	17 (9.1%)	*p* = 0.2384

## Data Availability

Data are available for consultation in any moment.
